# Der unbestimmte Ort des Eigentums: Ein theoretischer Zugang zum ambivalenten Verhältnis von Enteignung und demokratischer Rechtsstaat

**DOI:** 10.1007/s11615-021-00364-5

**Published:** 2021-12-22

**Authors:** Max Klein

**Affiliations:** Lehrstuhl für Politikwissenschaft/Politische Theorie, Philosophisch-Sozialwissenschaftliche Fakultät, Universität Augsburg, Augsburg, Deutschland

**Keywords:** Staatstheorie, Demokratietheorie, Weimarer Republik, Carl Schmitt, Otto Kirchheimer, State Theory, Democracy Theory, Weimar Republic, Carl Schmitt, Otto Kirchheimer

## Abstract

Eine zunehmende politische Infragestellung der bestehenden Privateigentumsordnung ist gegenwärtig zu konstatieren. Mit ernstzunehmenden Enteignungsforderungen auch außerhalb marginalisierter Splittergruppen kehrt ein Begriff zurück, der weitestgehend aus dem Blickfeld der Politischen Theorie verschwand. Der vorliegende Beitrag zielt in diesem Zusammenhang auf eine dezidiert politikwissenschaftliche Untersuchung des Rechtsinstituts der Enteignung, indem der Begriff in der Architektur des demokratischen Rechtsstaates verortet wird. Changierend zwischen den Polen der rechtsstaatlichen Garantie des Privateigentums einerseits und einer Potenzialität demokratischer Infragestellungen der Eigentumsordnung andererseits wird deutlich gemacht, dass der Enteignungsbegriff auf herausgehobene Weise die Aporien des demokratischen Rechtsstaates sichtbar macht. Die These wird durch eine theoriegeschichtliche Konturierung am Beispiel der intensiven Enteignungsdiskussionen in der Weimarer Republik und insbesondere mit tiefergehender Untersuchung der im Weiteren als Antipoden figurierten Positionen Carl Schmitts und Otto Kirchheimers dargelegt. Während Schmitt mit einem engen Rechtsstaatsbegriff eine weitgehende Zurückweisung von Enteignungspotenzialitäten zu plausibilisieren sucht, stellt Kirchheimer auf eine extensive Interpretation demokratischer Verfügungsgewalt über die Privateigentumsordnung ab. Die Weimarer Krisenjahre werden als eine Art „Laboratorium“ befragt, um vom außergewöhnlichen Maß kriseninduzierter Suchbewegungen zu profitieren und eine politikwissenschaftliche Theoriesprache für gegenwärtige Enteignungsdiskussionen in Ansätzen herauszuarbeiten.

## Abstract (en)

An increasing political contestation of the existing private-property order can currently be observed. With serious demands for expropriation, even outside marginalized splinter groups, a concept is returning that has largely disappeared from the focus of political theory. In this context, the article aims at a decidedly political–scientific examination of the legal institution of expropriation by locating the concept in the architecture of the democratic rule of law. Shifting between the poles of the constitutional guarantee of private property on the one hand and a potentiality of democratic contestation of the property order on the other, it is made clear that the concept of expropriation highlights the aporias of the democratic rule of law. The thesis is presented by means of a theoretical–historical contouring using the example of the intensive discussions on expropriation in the Weimar Republic and, in particular, with a more in-depth examination of the positions of Carl Schmitt and Otto Kirchheimer, which are subsequently figured as antipodes. While Schmitt seeks to make plausible a far-reaching rejection of expropriation potentials with a narrow concept of the rule of law, Kirchheimer focuses on an extensive interpretation of democratic power of disposition over the private-property order. The Weimar crisis years are examined as a kind of “laboratory” in order to profit from the extraordinary degree of crisis-induced searching and to work out a political science theoretical language for contemporary discussions of expropriation.

## Einleitung

Die Enteignung ist als politischer Begriff zurück. Unterlief sie bislang entweder aufgrund verwaltungsrechtlicher Unscheinbarkeit oder ideologischer Überschattung weitestgehend die Aufmerksamkeitsschwelle der meisten politischen Akteurinnen^1^ und der Politischen Theorie, wird gegenwärtig durch eine zunehmende politische Infragestellung der Privateigentumsordnung die Vermessung der Möglichkeiten, Reichweiten und Grenzen des Begriffs virulent: So irritieren Bürgerinnenbewegungen wie *Deutsche Wohnen & Co enteignen* die als selbstverständlich angenommene Eigentumsordnung durch Forderungen nach bezahlbarem Wohnraum, während das Bundesverfassungsgericht den *Berliner Mietendeckel* kassierte und dadurch den zugrundeliegenden politischen Konflikt gerade auch im nationalen Kontext noch einmal antrieb. Vor einen erweiterten Problemhorizont gestellt, offenbaren die hinter den Enteignungsforderungen stehenden Ansprüche insbesondere dann eine bedenkenswerte Pointe, wenn die sich sukzessive verschärfende ordnungspolitische Kontestation durch autoritäre Ordnungen mitberücksichtigt wird, die anders als konsolidierte liberale Demokratien westlicher Provenienz auch gesamtgesellschaftlich signifikante Wohlstandszuwächse vorweisen können (vgl. Schäfer und Zürn 2021, S. 9). Die Covid-19-Pandemie beschleunigt noch die sich bereits abzeichnenden Dynamiken. Kehrt der Enteignungsbegriff insofern als politische Kategorie zurück, wird eine politiktheoretische Untersuchung unumgänglich, die in der gegenwärtigen Politischen Theorie bisher auf eigentümliche Weise ausblieb^2^ und von der vorliegenden Studie geleistet werden soll.

Aus politischer Perspektive ist der Enteignungsbegriff vor allem deshalb interessant, da er auf eindrucksvolle Weise die Aporien des demokratischen Rechtsstaates sichtbar macht. Zum einen ist der demokratische Rechtsstaat als *liberaler Rechtsstaat* ein auf einem normativen Individualismus gegründeter und das individuelle Eigentum garantierender Staat und hat somit sozioökonomische Ungleichheiten begrifflich angelegt (vgl. beispielsweise Honneth 2017, S. 129–146, insbesondere S. 133–134). Zum anderen adressiert der Begriff des Demokratischen die Forderungen nach Formen von Egalität: Ein demokratischer Begriff der Bürgerin verweist auf eine Form anerkennender Gleichheit als Ermöglichung von Partizipationsfähigkeit (vgl. Seubert 2013, S. 25–26). Der demokratische Rechtsstaat beschwört damit theoretische Spannungen herauf, die zwischen theorieimmanenter materieller Ungleichheit – im Begriff des Rechtsstaates – und Forderungen nach Egalität – im Begriff des Demokratischen – changieren und in den gegenwärtigen Krisen deutlich intensiviert werden. Eine begriffliche Dominanz des Rechtsstaatlichen fordert insofern eine andere Reichweite der Enteignung als eine Vorrangigkeit des Demokratischen, da sie jeweils unterschiedlich – vollumfassend schützend oder sozial kontextualisierend – das Privateigentum politisch perspektivieren. Mit anderen Worten: Der Enteignungsbegriff wird zum Korrelat des Eigentumsbegriffs und verändert seine Konturen durch einen Wandel des Eigentumsverständnisses, das maßgeblich von der jeweiligen Ausdifferenzierung der politischen Ordnung bestimmt wird.

Die dargestellte Aporie des demokratischen Rechtsstaates soll im Folgenden an den Enteignungsdiskussionen in der Weimarer Republik theoriegeschichtlich konturiert werden. Die „Republik ohne Gebrauchsanweisung“, wie Alfred Döblin (1972, S. 100) die erste deutsche Demokratie nannte, bietet sich vor allem deshalb als Untersuchungsgegenstand an, da die Weimarer Revolution unmittelbar die Frage nach dem Neuen der aus der Revolution hervorgegangen Ordnung, dem Demokratischen, aufwarf und so ein beachtliches Maß theoretischer Innovationen generierte (vgl. Llanque 2008, S. 415). Aufgrund strukturell ähnlicher theoretischer Probleme könnten die Debatten Weimars für die Gegenwart fruchtbar gemacht werden. Ausgangspunkt der Überlegungen ist die Erschütterung der tradierten Begriffskulturen des Kaiserreichs im Zuge der revolutionären Transformation zum demokratisch-bürgerlichen Rechtsstaat der Weimarer Republik. Zum einen folgte die Weimarer Reichsverfassung weiterhin in vielen Teilen einem normativen Individualismus aus der Rationalität des liberalen 19. Jahrhunderts. Zum anderen kontestierte das Neue im Begriff des Demokratischen die konstitutionellen Deutungsmuster, sodass eine grundsätzliche Vermessung der Ordnung primäre Aufgabe der politischen Theoriebildung wurde. Die permanente Krisenhaftigkeit der Republik verschärfte zudem die ordnungstheoretischen Aporien und erzwang ein grundsätzliches Denken, das aufgrund seiner geradezu idealtypischen Überspitzung für eine Konturierung gegenwärtiger Probleme als eine Art „Laboratorium“ (vgl. Münkler 2006) firmieren kann.

In einem besonderen Maß geriet das Privateigentum in den *Krisenjahren der Klassischen Moderne* (Peukert 2018) unter einen einzigartigen Rechtfertigungsdruck, da im Zuge von Verelendung und gesellschaftlicher Desintegration die Frage nach den Reichweiten einer (grundlegenden) Umstrukturierung der Privateigentumsordnung nicht bloß in der Sphäre theoretischer Reflexion zirkulierte. Die aporetische Spannung des demokratischen Rechtsstaates fand in den Weimarer Debatten um das Privateigentum ihren anschaulichen Höhepunkt: Mit der Frage nach den materiellen Voraussetzungen der Demokratie war eine der wesentlichen Topoi des sozialistischen Denkens Weimars adressiert, das vor dem Hintergrund demokratietheoretischer Überlegungen auf eine materielle Integration der Arbeiterinnen drängte. Dass gerade bürgerliche Autorinnen zur Gegenseite tendierten und in der Kontinuität des liberalen normativen Individualismus stehend für einen umfassenden Privateigentumsschutz eintraten, kann als komplementäre Kompensation gedeutet werden. Insbesondere in theoretischen Suchbewegungen nach einer geeigneten Lesart der Enteignungsbestimmungen, an denen sich weitestgehend alle heute kanonisierten Figuren^3^ der Weimarer Staatswissenschaften^4^ beteiligten, wurden die eigentumstheoretischen Fragen der neuen Ordnung intensiv verhandelt. Skizzenhaft wird daher die Aporie des demokratischen Rechtsstaates im Folgenden an den Enteignungspositionen Carl Schmitts und Otto Kirchheimers dargestellt. Sowohl Schmitt als auch Kirchheimer leisteten paradigmatische Beiträge zur zeitgenössischen Debatte und können hinsichtlich ihrer jeweiligen Positionierung zum Enteignungsbegriff in Weimar idealtypisch als Antipoden figuriert werden. Während Schmitt mit seinem Begriff des bürgerlichen Rechtsstaates musterhaft in der Kontinuität des rechtsstaatlichen Paradigmas denkt, fokussiert Kirchheimer den Bruch mit dem Hinweis auf das Revolutionäre und folglich Demokratische. Die Gegenüberstellung dient als eine Art Heuristik, die Konfliktkonstellationen eines politiktheoretisch perspektivierten Enteignungsbegriffs sondiert.

Die Studie geht in drei Schritten vor. In einem ersten Teil wird die Weimarer Republik hinsichtlich ihrer spezifischen Sonderstellung zwischen Kontinuität des Rechtsstaatlichen und Neuanfang des Demokratischen ausgeleuchtet, um die ordnungspolitischen Antinomien des Weimarer Staates begrifflich für die weitere Untersuchung darstellbar zu machen. In einem zweiten Schritt wird vor diesem Hintergrund das Privateigentum genealogisch in seiner Veränderung vom 19. zum 20. Jahrhundert ausgeleuchtet, um einen Zugang zu den relevanten Fragen der Enteignungsdebatte zwischen Kirchheimer und Schmitt zu eröffnen. Abschließend werden erst Schmitt und dann Kirchheimer als Antipoden innerhalb des heuristischen Musters *Rechtstaat – Demokratie* ausdifferenziert und aufeinander bezogen reflektiert.

## Die Weimarer Revolution zwischen Neuanfang und Kontinuität

Die zeitgenössische Bewertung der Weimarer Revolution oszilliert zwischen Neuanfang und Kontinuität. Wurde sie zum einen als Aufbruch in ein demokratisches Zeitalter qualifiziert, musste die junge Republik doch zugleich auf das habituell etatistische, mindestens demokratieskeptische Beamtentum des Kaiserreichs zurückgreifen, um den Herausforderungen der politischen Neuordnung, der Friedensherstellung und gesellschaftlichen sowie wirtschaftlichen Integration im demoralisierten Deutschland verantwortungsvoll zu begegnen. Hatte mit der SPD nun die im Kaiserreich diffamierte Vertretung der Arbeiterinnenklasse die Möglichkeit, maßgeblich im Interesse der Arbeiterinnen auf die Gestaltung der zukünftigen Ordnung hinzuwirken, war es ebenso sehr aus der Perspektive einer Ordnungsrationalität geboten, die Interessen des Bürgertums und der (ehemaligen) Eliten konstitutionell zu berücksichtigen, um zivile Konfliktkanalisierungen dauerhaft zu ermöglichen (vgl. dazu Gusy 1997, S. 21–35). Unter dem Eindruck der gewaltsamen Folgen der Oktoberrevolution stehend galt es von einer Räteverfassung abzusehen und das Bürgertum konstruktiv zu integrieren, um die Gefahr eines schwelenden Bürgerkrieges abzuwenden (vgl. Böckenförde 2019b, S. 322; Winkler 2019, S. 68). In seinem zeitgenössischen Standardkommentar der Weimarer Reichverfassung schreibt daher Gerhard Anschütz (1926, S. 13):

Sie [die Mehrheitssozialdemokratische Partei Deutschlands; M. K.] trat vor allem – hierin mit den bürgerlichen Parteien einig – für die spezifisch demokratische Forderung ein, daß die Entscheidung über die künftige Staatsform und Verfassung Deutschlands nur von einer auf breiter Grundlage und unter Gleichberechtigung aller Gesellschaftsklassen gewählten, souverän beschließenden Nationalversammlung ausgehen könne.

Das mit der Verfassung ins Werk gesetzte Staatsgebilde des demokratisch-parlamentarischen Rechtsstaates war das Produkt der zwischen Vergangenem und Zukünftigem changierenden Revolution und avancierte zur Verdichtung des Klassenkompromisses. Zum einen schloss die neue Ordnung mit ihrer rechtsstaatlichen Verfasstheit an die Tradition des Konstitutionalismus an: Weiterhin war der Weimarer Staat von einem normativen Individualismus geleitet, der die rechtlich geschützte negative Freiheit der Einzelnen und ihr Privateigentum in das Zentrum seiner Rationalität rückte und damit dem Interesse des sich über Besitz und Bildung definierenden Bürgertums Rechnung trug. Ausdruck dieser Dichotomie von Staat und Individuum zur Realisierung des normativen Individualismus sind in der gewaltenunterscheidenden Konstitution, die durch ein balancierendes Misstrauenssystem willkürliche Staatseingriffe in die individuelle Freiheitssphäre ausschließen und die Staatsgewalt an das *Prinzip der Gesetzmäßigkeit der Verwaltung* binden soll, und den verfassungsrechtlich kodifizierten individuellen Grundrechten zu sehen. „Selbsterfüllung der individuellen Subjektivität als Sinn staatlich-öffentlicher Ordnung“ galt weiterhin als staatstheoretischer Nukleus (Böckenförde 2019a, S. 157). Prägnant heißt es daher in Ernst Rudolf Hubers (1981, S. 33) fulminanter Verfassungsgeschichte: „Der zu diesem Verfassungskompromiß bereite Teil der bürgerlichen Kräfte konnte an Vieles anknüpfen, was in der vorausgegangenen Epoche des Verfassungsstaates verwirklicht oder vorbereitet war.“

Zum anderen provozierte die Attribuierung des Demokratischen ein Verfassungsdenken, das über die bloße Individualität der bürgerlichen Freiheit hinausging und mit der Frage nach dem maßgeblichen Subjekt des Volkes zugleich die Frage nach den gesellschaftlichen Voraussetzungen demokratischer Herrschaftsordnung adressierte. Während der normative Individualismus der bürgerlichen Rechtsstaatsrationalität die Ungleichheit durch die Ermöglichung von Individualität als Unterschiedenheit zum Programm erhebt, fordert eine demokratische Praxis die Egalität der am politischen Prozess Partizipierenden. Anders als bürgerliche Abwehrrechte, die die abstrakte Rechtsgleichheit als Bedingung der Möglichkeit von individueller Differenz institutionalisieren, bedarf die Demokratie immer einer Art aktiver, das heißt sich politisch materialisierender Gleichheit, um gesellschaftliche Konflikte nicht im Medium staatlich angeleiteter paternalistischer Distributionsverfahren zu lösen, sondern in Form politischer Artikulation von differierenden gesellschaftlichen Interessen auf zivilem Weg zu kanalisieren. In Weimar wurde daher nicht allein die erstmals eingeführte formal-individualistische Kategorie des gleichen Wahlrechts ventiliert, sondern darüber hinausgehend ebenso sehr die politische Integration der Arbeiterinnenklasse durch die Befragung materieller und/oder ideeller Grundvoraussetzungen eines für das Gelingen demokratischer Ordnungen wesentlichen Kollektivbewusstseins verhandelt (vgl. Llanque 2015). Der Begriff der Demokratie war maßgeblich eine Integrationschiffre, wie Hermann Heller (1992b, S. 289; vgl. auch 1992a, S. 423) hervorhebt: „Nachdem das Bürgertum die Mitherrschaft wenigstens dem Grundsatz nach erlangt, geht die demokratische Forderung an den vierten Stand, an das Proletariat über.“

Wird daran anschließend eine Verfassung als geronnene Politik perspektiviert (vgl. dazu Groh 2010, S. 79), mag es kaum verwundern, dass gerade der bereits von Zeitgenossinnen häufig geschmähte Zweite Hauptteil der Weimarer Reichsverfassung, *Grundrechte und Grundpflichten der Deutschen*, als unzureichendes Kompositum unterschiedlichster Programmsätze gedeutet wurde (vgl. dazu Stolleis 1999, S. 90, 109–114). Der als „interfraktionelles Parteiprogramm“ (Pauly 2004, S. 1) diffamierte Grundrechtskatalog übersetzte vor allem in Wirtschaftsfragen die Dichotomie von Bürgertum und Arbeiterinnenklasse in eine duale Struktur, die in einem Mit- und Nebeneinander von liberalen Abwehrrechten und sozialen Programmsätzen zum Ausdruck kam.^5^ Im *Grundrechtslaboratorium Weimar* (Pauly 2004) trafen klassisch liberale Grundrechte wie „Unverletzlichkeit der Wohnung“ (Artikel 115 WRV), „Gleichheit vor dem Gesetz“ (Artikel 109 WRV) und „Recht der freien Meinungsäußerung“ (Artikel 124 WRV) auf anspruchsvolle Zukunftsprojekte wie den „Sozialisierungsgesetzen“ (insbesondere Artikel 155 und 156 WRV). Vor allem das Grundrecht auf Privateigentum nimmt in dieser bipolaren Heuristik einen neuen Charakter an: Es ist weder allein Abwehrrecht noch gänzlich der demokratischen Verfügungsgewalt unterworfen. Dies macht eine genealogische Perspektivierung deutlich.

## Der unbestimmte Ort des Eigentums im demokratischen Rechtsstaat der Weimarer Republik

Für den im 19. Jahrhundert virulent werdenden Begriff des *bürgerlichen* Rechtsstaates gilt das Privateigentum als wesentliche legitimatorische Säule. Die Staats- und Rechtsphilosophie Immanuel Kants ist hinsichtlich der spezifischen Legitimationsstruktur eines Staatsbegriffs dieser Art paradigmatisch. Kants in diesem Zusammenhang entscheidende Schrift *Die Metaphysik der Sitten *greift in ihrer Grobstruktur bereits die argumentative Stoßrichtung moderner Rechtskonzeptionen auf: Erst führt er das Privatrecht ein und lässt dann das Öffentliche Recht resultieren. Entscheidend ist, dass Kant seine Rechtslehre damit nicht auf der Grundlage einer objektiven Gerechtigkeitskonzeption plausibilisiert, sondern vielmehr das Öffentliche Recht über das Privatrecht herleitet und so die für modernes Recht typische Normstruktur reproduziert. Anstatt die Legitimation des Rechts über Verweise an metaphysisch-theologische Semantiken darzulegen, gründet das Rechtssystem auf der individuellen Willkür der Einzelnen – auf der bürgerlichen Freiheit. Mit Christoph Menke (2018, S. 31; Hervorhebungen im Original) gesprochen, ist hier die Revolution des modernen Rechts sichtbar, indem sich die „*Funktionsbestimmung des Rechts überhaupt*“ wandelt: „Das Recht ist jetzt *dazu da*, Rechte zu sichern.“ Der Primat des Anspruchs strukturiert die Logik des Rechts.

Das Privatrecht seinerseits beginnt bei Kant mit dem „äußeren *Mein* und *Dein* überhaupt“ (Kant 1969, S. 245; Hervorhebungen im Original). So heißt es im Privatrechtskapitel: „Das rechtliche Meine (*meum iuris*) ist dasjenige, womit ich so verbunden bin, daß der Gebrauch, den ein anderer ohne meine Einwilligung von ihm machen möchte, mich lädieren würde“ (Kant 1969, S. 245; Hervorhebungen im Original). Das hier beschriebene und für das Privateigentum konstitutive, ja den Begriff selbst bestimmende Exklusionsrecht ist der Ausdruck einer von Kant vorgenommenen moralischen Qualifizierung des individuellen Eigentums, indem er die Aneignung von „äußeren Gegenständen“ mit einem Begriff der individuellen Freiheit verbindet, die individuelle Freiheit damit zur inneren Norm des Eigentums erhebt (vgl. auch Wesche 2014, S. 451), und ferner mit der praktischen Vernunft vereinbar sieht: „Es ist möglich, einen jeden äußeren Gegenstand meiner Willkür als das Meine zu haben; d. i.: eine Maxime, nach welcher, wenn sie Gesetz würde, ein Gegenstand der Willkür an sich (objektiv) herrenlos (*res nullius*) werden müßte, ist rechtswidrig“ (Kant 1969, S. 246; Hervorhebungen im Original). Kants Exklusionsrechtfertigung reproduziert auf diese Weise das bereits bei Locke aufgegriffene und bei Hegel moralisch erweiterte Legitimationsschema des Staates: Wenn der bürgerliche Rechtsstaat Garant individuell-negativer Freiheit ist und das Privateigentum als materielle Bedingung der Möglichkeit einer Verobjektivierung ebendieser Freiheit verstanden wird, ist eine der wesentlichen Legitimationsgründe in dem Schutz individuellen Eigentums zu sehen.^6^

Historisch materialisierte sich diese Form privatrechtlicher Rechtsbegründung in Deutschland mustergültig im Eigentumsartikel der Preußischen Verfassung von 1850. Im Artikel 9 wird der Kern rechtsstaatlichen Verfassungsdenkens konzentriert zum Ausdruck gebracht: „Das Eigenthum ist unverletzlich. Es kann nur aus Gründen öffentlichen Wohls gegen vorgängige, in dringenden Fällen wenigstens vorläufig festzustellende Entschädigung nach Maaßgabe des Gesetzes entzogen oder beschränkt werden.“ Strukturhomolog zur kantischen Argumentation wird das Eigentum als negative Rechtssphäre dem Zugriff des Staates prinzipiell entzogen und in die private Willkürsphäre der Einzelnen verlagert; das Eigentum wird als Abwehrrecht des Individuums gegenüber der Willkür des Staates plausibilisiert, das heißt, der Staat findet am Privateigentum und somit an der rechtlich sanktionierten Verfügungsgewalt der Einzelnen an Gegenständen seine Grenze. Carl Schmitt (2017, S. 158; Hervorhebungen im Original) hat in seiner Theorie der Grundrechte in der *Verfassungslehre* für das beschriebene Verhältnis von Individuum und Staat im bürgerlichen Rechtsstaat wirkmächtig den Begriff des *Verteilungsprinzips* vorgeschlagen:

Für die systematische Betrachtung des modernen Rechtsstaates handelt es sich darum, daß der Gedanke der Grundrechte das fundamentale Verteilungsprinzip enthält, auf welchem der konsequent durchgeführte bürgerlich-freiheitliche Rechtsstaat beruht. Das bedeutet, daß die Freiheitssphäre des einzelnen prinzipiell *unbegrenzt*, die Befugnisse des Staates prinzipiell *begrenzt* sind.

So wie exemplarisch an Kants Staatsphilosophie beschrieben, ist die Struktur des Rechts durch ein Theorem individueller Ansprüche begründet. Die Chiffre der Unverletzlichkeit des Eigentums ist die verfassungsrechtliche Kodifikation eines Appells an die Staatsgewalt, die normative Vorrangigkeit des individuellen Anspruchs in Gestalt subjektiver Rechte anzuerkennen.

Dass Artikel 9 Satz 2 trotz des emphatischen Unverletzlichkeitsindikativs dennoch Entziehung oder Beschränkung „aus Gründen öffentlichen Wohls […] nach Maaßgabe des Gesetzes“ zulässt, mag paradox wirken, wird allerdings durch den Verweis auf eine Entschädigungspflicht relativiert. Der Enteignungsvorbehalt, der in dem Artikel zum Ausdruck kommt, scheint vordergründig dem Unverletzlichkeitspostulat zu widersprechen, offenbart sich allerdings durch die Entschädigungsnotwendigkeit nicht als Eigentumsnegation, sondern im Gegenteil als Eigentumsaffirmation: Der Eingriff in das Privateigentum verpflichtet zur Entschädigung, die Ausdruck einer Anerkennung der Rechtmäßigkeit der Eigentümerin an ihrem enteigneten Eigentum ist, und affirmiert damit die bestehende Privateigentumsordnung. Dieses – in der Sprache des Verwaltungsrechts auch als *klassischer Enteignungsbegriff* bezeichnetes – Verhältnis von „Anerkennung und Entziehung“ (Schmitt 1973, S. 120) wurde bereits bei Otto Mayer (1969, S. 30), der Gründungsfigur des deutschen Verwaltungsrechts, in seinem *Deutschen Verwaltungsrecht* von 1895 klar beschrieben:

Die Enteignungsentschädigung ist nichts anderes als die besondere Anwendung, allerdings zugleich auch die hervorragendste, eines allgemeineren Billigkeitsgrundsatzes, wonach der einzelne, dem zugunsten der öffentlichen Verwaltung ein besonderes Opfer zugemutet werden mußte, von dieser Verwaltung einen Ausgleich in Geld erhalten soll.

Der Artikel 9 der Preußischen Verfassung veranschaulicht dadurch in seiner spezifischen Eigentumsgarantie den zuvor beschriebenen normativen Individualismus in einer besonderen Klarheit, da er die negative Freiheitssphäre durch die Institutionalisierung eines privaten Verfügungsraums über die materielle Selbstorganisation prinzipiell sicherstellt beziehungsweise den Eingriff in diese als rechtfertigungsbedürftiges und entschädigungsnotwendiges Verfahren qualifiziert.

Die bereits beschriebenen Irritationen der tradierten Begriffskulturen durch die Weimarer Revolution zeitigen gerade mit Blick auf das Grundrecht des Privateigentums eine wesentliche Verschiebung hinsichtlich der rechtsstaatlichen Eigentumsgarantie. Offensichtlich ist, dass der Unverletzlichkeitsindikativ des Privateigentums und eine unumgängliche Entschädigungsnotwendigkeit eine Garantie des proprietären Status quo verfassungsrechtlich überführt. Dass die Weimarer Reichsverfassung als Ausdruck des Klassenkompromisses einen anderen Weg gehen musste, scheint wenig erklärungsbedürftig: Aus der Sicht der Arbeiterinnenklasse und ihrer politischen Vertretung wäre die verfassungsrechtliche Zementierung der Eigentumsordnung eine einseitige Entscheidung für die Interessen des Bürgertums. Die Irritation des bürgerlichen Rechtsstaates durch das Demokratische rief vielmehr eine Infragestellung der bestehenden Eigentumsordnung auf den Plan, da demokratietheoretisch mit der Forderung nach Egalität – wie oben bereits angesprochen – mehr als bloß eine Hoffnung auf formale Gleichheit mitschwang (vgl. Heller 1992a, S. 427).^7^ Mit dem Begriff des Demokratischen nimmt das Privateigentum nun zumindest einen unbestimmten Ort in der Architektur des demokratischen Rechtsstaates ein: Der bürgerliche Rechtsstaat fordert die Eigentumsgarantie im Medium des bürgerlichen Rechts, während die Demokratie eine Infragestellung des Eigentums zur Herstellung eines einheitlichen Willens zumindest als *Möglichkeit* aufwirft (vgl. ähnlich Colliot-Thélène 2016).

Im Artikel 153 WRV, dem „Kernstück des Weimarer Wirtschaftsverfassungsrechts“ (Huber 1981, S. 113), nimmt die Unbestimmtheit des Eigentums im demokratischen Rechtsstaat Verfassungsrang an:

Das Eigentum wird von der Verfassung gewährleistet. Sein Inhalt und seine Schranken ergeben sich aus den Gesetzen.

Eine Enteignung kann nur zum Wohle der Allgemeinheit und auf gesetzlicher Grundlage vorgenommen werden. Sie erfolgt gegen angemessene Entschädigung, soweit nicht ein Reichsgesetz etwas anderes bestimmt. Wegen der Höhe der Entschädigung ist im Streitfalle der Rechtsweg bei den ordentlichen Gerichten offen zu halten, soweit Reichsgesetze nichts anderes bestimmen. Enteignung durch das Reich gegenüber Ländern, Gemeinden und gemeinnützigen Verbänden kann nur gegen Entschädigung erfolgen.

Eigentum verpflichtet. Sein Gebrauch soll zugleich Dienst sein für das Gemeine Beste.

Anders als die Preußische Verfassung von 1850 ist das Eigentum in der Weimarer Reichsverfassung nicht mehr unverletzlich, sondern wird erst von der „Verfassung gewährleistet“; Schranken und Inhalt ergeben sich aus den Gesetzen. Während die Eigentumsgarantie im bürgerlichen Rechtsstaat als Imperativ *gegen* den Staat eher ein Kontrakt zwischen Bürgertum und Staatsgewalt war, avanciert der Staat in Weimar vielmehr zur notwendigen Bedingung des Eigentums. Das Verhältnis verkehrt sich: Das Privateigentum ist kein Abwehrrecht gegenüber dem Staat, sondern wird von diesem gewährt; es entsteht keine bürgerliche Hermetik gegenüber der Staatssphäre, sondern die Sphäre des Staates *wird* der Ursprung des Eigentums, sodass dieser dauerhaft und vor allem nicht allein negativ auf das Privateigentum rückbezogen bleibt. Gerade deshalb bindet die Weimarer Reichsverfassung durch den Absatz 3 das Privateigentum auch an soziale Kompatibilität – „Eigentum verpflichtet“ – und verlagert die Verfügungsgewalt nicht mehr allein in die Willkürsphäre der Einzelnen, sondern politisiert die Privatheit des Eigentums hinsichtlich seiner sozialen Ausrichtung.

Die entscheidende Neuerung besteht vor allem in der Veränderung der verfassungsrechtlichen Enteignungsmöglichkeiten, die im Zusammenhang mit der Politisierung des Eigentums ebenso eine soziale Wendung implizieren. Im Modus des klassischen Enteignungsrechts bindet zwar auch die Weimarer Reichsverfassung die Entziehung des Privateigentums an dessen Anerkennung qua Entschädigung. Andererseits spielt sie den Verdacht eines weiteren Enteignungsbegriffs ein, wenn sie hinzufügt, dass eine Entschädigung ausbleiben könne, soweit „ein Reichsgesetz etwas anderes bestimmt“. Die kategorische Abhängigkeit von Entzug und Anerkennung wird damit aufgelöst und die Potenzialität eines Enteignungsbegriffs, der den Status quo der Privateigentumsordnung infrage stellt, aufgerufen. Präzise schreibt daher Walter Jellinek (1928, S. 395) in seinem *Verwaltungsrecht* von 1928 über die Enteignungspotenzialitäten in der Weimarer Reichsverfassung:

Die Enteignung bedarf endlich nicht immer des Zwischenglieds eines Verwaltungsaktes, kann vielmehr auch kraft Gesetzes eintreten. Dabei sind zwei Möglichkeiten gegeben: allgemeines Gesetz und Gesetz für den Einzelfall. […] Enteignungsgesetze für den Einzelfall sind zwar wenig rechtsstaatlich, aber durch die Reichsverfassung, die den Montesquieuschen Gedanken keineswegs zum Verfassungssatze erhoben hat, nicht verboten.

Jellinek beschreibt mit dem Hinweis auf die „zwar wenig rechtsstaatlich[e]“ Praxis der entschädigungslosen Enteignung durch Einzelfallgesetze die mit dem Begriff des Demokratischen aufgerufene Abweichung von einem Idealtyp des bürgerlichen Rechtsstaates und dessen klassischen Enteignungsbegriffs. Die Politisierung des Privateigentums durch die Rückbindung an die Sozialkompatibilität scheint damit das Muster der demokratischen Irritation des bürgerlichen Rechtsstaates zu bestätigen. Der Klassenkompromiss konzentriert im Artikel 153 deutlich die ihn bestimmenden politischen Spannungen, indem zwar das „Privateigentum unter den Schutz und die Garantie der Verfassung“ (Anschütz 1926, S. 396) gestellt, allerdings durch die neue Formulierung zunehmend plastisch wird. Die Ambivalenz des Artikels 153 ist der rechtliche Ausdruck des tieferliegenden politischen Konflikts, der zwischen den Forderungen einer individualistischen negativen Freiheitskonzeption und dem Egalitätsanspruch des Demokratischen eine rechtsimmanente Lösung versperrt und im Medium der Verfassung die hinter der Dichotomie stehende Frage zurück an das Politische spielt.

## Enteignung als rechtspolitischer Begriff – Carl Schmitt und Otto Kirchheimer

Die Ambivalenz im Enteignungsrecht provozierte sowohl bürgerliche als auch sozialistische Akteure, die Richtung der Republik durch das Interpretieren des Artikels 153 in ihrem Sinn zu bestimmen. Auch wenn Verfassungsrecht aufgrund seiner geringen Normdichte immer umstrittenes Recht bleibt, nahm die ordnungstheoretische Unbestimmtheit des Artikels 153 in Weimar eine politische Intensität an, die über gewöhnliche Auslegungskonflikte hinausging. Intensiviert und emotionalisiert wurde die Debatte von einem sich ausbreitenden kriseninduzierten Evidenzverlust des Privateigentums im Zuge von Verelendungsprozessen; die enormen wirtschaftlichen Krisen nötigten zu einer grundlegenden Ausmessung der Reichweiten einer Politisierung des Privateigentums und ließen die ordnungstheoretischen Aporien der Ersten Republik mit Vehemenz auf den Plan treten. Es ist daher kaum verwunderlich, dass gerade die Diskussionen um das Enteignungsrecht mehr polemisch-legitimatorischen als analytischen Charakter annahmen. Die in Weimar virulent gewordene Aporie im Begriff des demokratischen Rechtsstaates übersetzt sich in den politisch motivierten differierenden Interpretationen der Verfassung, die den Kern des bestehenden Staates selbst berührten.

Die Hauptfrage, ob der demokratische Rechtsstaat Weimars primär das Demokratische oder das Rechtsstaatliche betont, ist dabei kein gradueller Unterschied, sondern ein qualitativer, da mit der Bestimmung des Ortes des Privateigentums im Wesentlichen das Verhältnis von Staat, Individuum und Demos adressiert und vollkommen unterschiedlich angeordnet wird. Aus einer politikwissenschaftlichen Perspektive ist demnach an Hegemoniepolitiken dieser Art interessant, dass sie stets vor dem Hintergrund relativ klarer staatstheoretischer Muster getroffen werden und diese erst die Plausibilität ihrer Deutungen konstituieren. Die Rechtsdeutung wird insofern nicht allein, ja nicht einmal primär juristisch hergeleitet, sondern am evaluativen Maßstab eines der Rechtsdeutung vorausgehenden Staatsbegriffs bemessen. Dies wird im Folgenden detailliert an Carl Schmitt und Otto Kirchheimer dargelegt, indem der spezifische Staatsbegriff, der den jeweiligen Argumentationen über die Zulässig- beziehungsweise Unzulässigkeit von entschädigungslosen Enteignungen zugrunde liegt, befragt wird.

### Carl Schmitt: Bürgerlicher Rechtsstaat, generelles Gesetz und Eigentumsgarantie

In seinem Hauptwerk *Verfassungslehre* von 1928 unternimmt Carl Schmitt (2017, S. XI) den „Versuch eines *Systems*“: „In der Hauptsache ist“, so Schmitt (2017, S. XIII) im Vorwort, „die Verfassungslehre des bürgerlichen Rechtsstaates dargestellt“. Der bürgerliche Rechtsstaat seinerseits sei „diese Art Staat“, die „heute im allgemeinen noch vorherrschend“ ist und die der „Weimarer Verfassung entspricht“ (Schmitt 2017, S. XIII). Vor dem Hintergrund des bisher Gesagten ist die Terminologie Schmitts vielsagend, da sie durch die Betonung des Bürgerlichen eine Kontinuitätsbeziehung zum Kaiserreich herstellt und so das Revolutionäre der ersten deutschen Demokratie bereits semantisch unterläuft. Anders als Autoren wie Kirchheimer, Anschütz und Franz Neumann spricht Schmitt nicht vom *demokratischen* Rechtsstaat, sondern greift mit der Dominanz des Bürgerlichen auf das Begriffsrepertoire der „Hochphase des Liberalismus im 19. Jahrhundert“ (Müller 2018, S. 21) zurück. Innerhalb der heuristischen Dichotomie von Demokratie und Rechtsstaat ordnet Schmitt (2017, S. 31; Hervorhebungen im Original) die Weimarer Reichsverfassung der Kontinuität des Rechtsstaatlichen zu: „Die Entscheidung [der WRV; M. K.] mußte für den bisherigen sozialen *status quo*, d. h. für die Beibehaltung der bürgerlichen Gesellschaftsordnung fallen […].“^8^

Der bürgerliche Rechtsstaat, so betont auch Schmitt, macht den Schutz und die Garantie der bürgerlichen Freiheit im Medium des Rechts zum Nukleus seiner Bestimmung (vgl. Schmitt 2017, S. 129). Unter Recht ist im bürgerlichen Rechtsstaat insofern keine beliebige rechtliche Norm ausgewiesen, sondern eine als subjektives Recht begriffene. Damit ist die negativ verstandene Freiheitssphäre des Individuums in den Mittelpunkt der Rationalität gerückt. In dem Begriff des Bürgerlichen dominiert folglich nicht die Vorstellung des „citoyen“ und mit ihm ein aktives Partizipationsrecht, sondern vielmehr der „bourgeois“ und somit ein von Sekuritätsüberlegungen geleitetes passives Abwehr- und Schutzrecht vor Formen des staatlichen Eingriffs vor allem in die individuelle Handlungssphäre und ökonomieförmige Selbstorganisation (vgl. Schmitt 2017, S. 168). Damit ist der Rechtsbegriff im bürgerlichen Rechtsstaat vor allem ein liberaler Begriff im Sinne einer theoretischen Vorrangigkeit des Individuums, wie er am Beispiel der Staats- und Rechtsphilosophie Kants oben dargestellt wurde.

Die rechtliche Abstraktion zum liberalen Subjekt führt zu einer allgemein-egalitären Struktur des bürgerlichen Rechts, die eine atomisierte Perspektive unabhängig von historischer Eingebundenheit vorgibt und somit beispielsweise Formen ständischer Privilegierung prinzipiell unterläuft. Das liberale Individuum ist paradoxerweise frei von jeglicher Individualität. Insofern erhält das abstrakte Individuum eine polemische Stoßrichtung, da es sich immer gegen Formen der historisch situierten Prägung und Setzung entgegenstellt. Der Verweis auf das allgemeine Individuum unterminiert konsequent jede Praxis personaler Herrschaft, da quasigedankenexperimentell ein Zustand der Egalität als normativer Ausgangspunkt evoziert wird. An diesen Gedankenkomplex anschließend führt Schmitt in *Der Begriff des Politischen* von 1932 aus: „Denn die Negation des Politischen, die in jedem konsequenten Individualismus enthalten ist, führt wohl zu einer politischen Praxis des Mißtrauens gegen alle denkbaren politischen Mächte und Staatsformen, niemals aber zu einer eigenen positiven Theorie von Staat und Politik“ (Schmitt 2015 [1932], S. 64). Die Einzelne als Gleiche unter Gleichen ist ein polemischer Begriff gegen menschliche Herrschaftsansprüche, der sich historisch vor allem gegen den absolutistischen Staat richtete. Der bürgerliche Rechtsstaat „enthält […] die Ablehnung der Herrschaft von Menschen, sei es eines einzelnen Menschen, sei es einer Versammlung oder Körperschaft, deren *Wille* an die Stelle einer für alle gleichen, im voraus bestimmten generellen Norm tritt“ (Schmitt 2017, S. 139; Hervorhebung im Original).

Da der Befehl und die widerspruchslose Autorität aus der Rechtsstaatsrationalität verdrängt werden, firmiert der bürgerliche Rechtsstaat als *Herrschaft des Gesetzes*: Alle Tätigkeit des Staates muss in eine „allgemeine[] *Meßbarkeit*“ übergehen (Schmitt 2017, S. 131; Hervorhebungen im Original). Das Gesetz wiederum wird durch seinen Zusammenhang mit der bürgerlichen Freiheit qualifiziert und im Kontrast zu Maßnahme und Befehl als generelle Norm ausbuchstabiert. Dass das Gesetz als *generelle* Norm ausgewiesen werden muss, ergibt sich aus dem Begriff des bürgerlichen Rechts selbst. Wenn das bürgerliche Recht den Schutz der negativen Freiheitssphäre der Einzelnen zur vorgelagerten Norm erhebt, fallen reale Unterscheidungen von Personen aus dem Blick. Ungleichheiten müssen verallgemeinert, getragen von nachvollziehbaren Gründen, gerechtfertigt werden, wenn das abstrakt-egalitäre Individuum als normativer Maßstab den Ausgangspunkt bildet. Das Gesetz des Rechtsstaates ist damit ein Schutz vor willkürlich-unbegründeten Formen des Eingriffs durch personale Herrschaft in die bürgerliche Freiheit (vgl. Schmitt 2017, S. 146).

Bereits in der Darstellung der kantischen Rechtsphilosophie wurde die Bedeutung des Privateigentums für den Begriff bürgerlicher Freiheit deutlich. Das Privateigentum ist die materielle Bedingung der Möglichkeit einer Verobjektivierung ebendieser Freiheit. Carl Schmitts Begriff des Privateigentums schließt an diesen Diskurs an – ohne Kant selbst als Referenz zu nennen – und bleibt weiterhin in den Bahnen einer konstitutionellen Deutungslinie^9^: Insofern das bürgerliche Recht als Übersetzung der bürgerlichen Freiheit der Nukleus der Bestimmung des Rechtsstaates ist, erhält das Eigentum im klassischen liberalen Sinn eine rechtliche Sonderstellung im Gefüge der Rechtsstaatsarchitektur. Dies wird erwartungsgemäß an Schmitts Interpretation des Artikels 153 deutlich. Indem Carl Schmitt den Weimarer Staat einseitig rechtsstaatlich überformt, ist es nur konsequent, dass er den Artikel 153 ausdrücklich in einer rechtsstaatlichen Deutungstradition perspektiviert und dezidiert eine Garantie des Privateigentums stark macht. Schmitt (2017, S. 171–172) gesteht zwar zu, der „Wortlaut“ des Artikels 153 sei „[w]iderspruchsvoll und unklar“, deutet ihn dann aber letztendlich vor dem Hintergrund der ordnungstheoretischen Prämissen des Rechtsstaates und harmonisiert die dem Eigentumsartikel inhärente Inkongruenz. So heißt es in der *Verfassungslehre* (2017, S. 172; Hervorhebungen im Original):

In Satz 1 [des Artikels 153, „Das Eigentum wird von der Verfassung gewährleistet“; M. K.] ist die Gewährleistung des Eigentums nicht als verfassungsgesetzliche Gewährleistung eines inhaltslosen Namens, sondern als Anerkennung eines Prinzips gemeint, weil es keinen bürgerlichen Rechtsstaat ohne Privateigentum geben kann und die Weimarer Verfassung eine bürgerlich-rechtsstaatliche *Verfassung* sein will.

Mit besonderer Klarheit kommt in dem Zitat das Verhältnis von Staatsverständnis und Eigentumsbegriff zum Ausdruck: Durch die rechtsstaatliche Ausdeutung des Weimarer Staates generiert Schmitt ein Rechtsstaatsparadigma, das durch die spezifische Konstellation von bürgerlicher Freiheit, bürgerlichem Recht, Staat und Gesetz das Privateigentum als Begriff der bürgerlichen Sphäre ausdifferenziert und dadurch als das dem Staat Entzogene konturiert. Staat und eine als Akkumulation von Individuen begriffene Gesellschaft werden in divergierende Sphären geteilt und die Sphäre des Bürgerlichen hermetisch dem Staat gegenübergestellt. Die Argumentation, die Schmitt hier bemüht, ist bereits vertraut: Das Privateigentum wird durch die *Anerkennung des Prinzips* im Sinne einer individuellen Verfügungsgewalt in die Privatsphäre der Einzelnen verlagert und dadurch als unpolitischer Begriff qualifiziert, da der Staat bloß das Prinzip, das heißt die Potenzialität, sich Eigentum anzueignen und darüber frei zu verfügen, anerkennt und sich folglich selbst exkludiert. Die soziale Dimension, die Artikel 153 Absatz 3 – „Eigentum verpflichtet“ – auf den Plan ruft, wird von Schmitt ignoriert.

Wird nun danach gefragt, welche Konsequenzen der beschriebene rechtsstaatliche Gesetzesbegriff für die Institution der Enteignung in der Weimarer Reichsverfassung hat, zeigt sich deutlich eine Engführung, die aus der spezifischen konstitutionalistischen Perspektivierung der Weimarer Reichsverfassung resultiert. Wie dargestellt wurde, ist der Artikel 153 vor allem in Hinblick auf die Enteignungspotenzialitäten unklar. Während unstrittig zu sein scheint, dass der klassische Enteignungsbegriff weiterhin durch das Verhältnis von Entziehung und Anerkennung qua Entschädigung Teil der Verfassungsordnung bleibt, ist zumindest die Möglichkeit durch den Hinweis einer entschädigungslosen Enteignung in der Form eines Reichsgesetzes impliziert. Schmitts spezifische Ausformung des Gesetzesbegriffs dient hierbei als Hebel, gerade diese Möglichkeit einer entschädigungslosen Enteignung kategorisch auszuschließen. So schreibt er in der *Verfassungslehre*:

Die rechtsstaatliche Garantie richtet sich gegen jeden Absolutismus und setzt jedem politischen Gesetzesbegriff, mag es nun der monarchische oder der demokratische sein, sachliche Schranken, indem sie verhindert, daß jeder vom Gesetzgeber, d. h. der für den Erlaß von Gesetzen zuständigen Stelle vorgenommene Eingriff in Freiheit und Eigentum als Gesetz betrachtet wird, vielmehr stets eine Norm mit bestimmten Qualitäten vorausgesetzt ist (Schmitt 2017, S. 150).

Schmitts Kontrastierung eines rechtsstaatlichen von einem politischen Gesetzesbegriff offenbart vor dem Hintergrund der gesetzlichen Sonderrolle des Privateigentums eine polemische Pointe: Da der rechtsstaatliche Gesetzesbegriff als Achse des Rechtsstaates ausdifferenziert wurde, werden Einzelfallgesetze als illegitimer Rückfall absolutistischer Herrschaftsäußerungen begriffen. Einzelfallgesetze, die unmittelbar enteignend auf die Privateigentumsordnung einwirken, sind im Zusammenhang mit dem Rechtsstaatsimperativ unzulässig, da sie den Egalitätsanspruch des rechtsstaatlichen Gesetzes missachten und im Modus des Gesetzgebungsverfahrens eine Deprivilegierung einer Personengruppe durch den gezielten Eingriff in ihr Eigentum vornehmen. Dadurch, dass Schmitt den Absolutismus im oberen Zitat nicht bloß als historische Kategorie ausdifferenziert, sondern als analytischen Begriff auf alle Herrschaftsformen ohne Gesetzesbindung erweitert, erhält er die Möglichkeit, die Weimarer Gesetzgeberin an den rechtsstaatlichen Egalitätsimperativ des rechtsstaatlichen Gesetzes zu binden und Enteignungen als spezifische Umverteilungsmaßnahmen juristisch zu disqualifizieren.^10^ In seinem für die Weimarer Enteignungsdebatte eminenten Text *Die Auflösung des Enteignungsbegriffs* von 1929 führt Schmitt (1973, S. 116) in diesem Sinn aus:

Es ist daran festzuhalten, daß der Schutz des Privateigentums durch Art. 153 grundsätzlich durch die Rechtslage des Jahres 1919 bestimmt ist. […] Der Schutz gegen den Gesetzgeber richtet sich nicht gegen generelle Bestimmungen, sondern nur gegen Mißbrauch der Gesetzgebungsform zu konkreten Enteignungsakten.

Der rechtsstaatliche Gesetzesbegriff wird damit ein Mechanismus zur Zurückweisung und Hemmung demokratischer Herrschaft und offenbart seine spezifische politische Stoßrichtung gegen sozialistische Ansprüche möglicher entschädigungsloser Enteignungspraktiken.

Da Enteignungen qua Einzelfallgesetz nicht mit der Generalität des Gesetzes vereinbar sind, können Enteignungsgesetze bloß Verfahren generieren, das heißt, Enteignung erfolgen allein *auf der Grundlage des Gesetzes* und nur durch* Gesetzesanwendung*: „Der Gesetzgeber soll eben nicht Prozesse entscheiden, sondern Gesetze geben, d. h. Rechtsregeln zur Entscheidung von Prozessen“ (Schmitt 1926, S. 10). Der Unterschied von Gesetzesgrundlage und -anwendung ist insofern entscheidend, als Schmitt auf diese Weise jede Form der Enteignung an die Entschädigungsnotwendigkeit rückbindet. In dem historisch äußerst wirksamen – und von der DNVP stark gemachten (Schüren 1978, S. 54) – Rechtsgutachten zu den Gesetzesentwürfen über die Vermögensauseinandersetzung mit den depossedierten deutschen Fürstenhäusern („Fürstenenteignung“) führt Schmitt 1926 diese Überlegungen weiter aus:

Artikel 153 spricht davon, daß eine Enteignung „nur auf gesetzlicher Grundlage“ vorgenommen werden kann. […] Wenn nun gesagt ist, daß eine Enteignung „nur auf gesetzlicher Grundlage“ vorgenommen werden kann, so bedeutet dies: der das konkrete Eigentum treffende, einzelne Eingriff setzt ein Gesetz voraus. Eine Enteignung in dem weiten Sinne des Artikels 153 darf mit anderen Worten nur ein Akt der Gesetzesanwendung sein. Der Unterschied von Gesetz und Gesetzesanwendung ist nicht etwa juristische Haarspalterei, sondern ein elementarer Grundbegriff sowohl der Lehre von der Gewaltenteilung als des modernen, rechtsstaatlichen Denkens; er gehört infolgedessen auch zum positiven Inhalt der Weimarer Verfassung (Schmitt 1926, S. 17–18).

Das Resultat dieser Argumentation ist, dass im Modus des klassischen Enteignungsbegriffs entschädigungslose Umverteilungsmaßnahmen grundsätzlich ausgeschlossen werden.

Schmitt legt vor diesem Hintergrund den Artikel 153 wesentlich enger aus als andere bürgerliche Juristen wie Walter Jellinek und Gerhard Anschütz, die zwar ähnlich wie Schmitt Einzelfallgesetze nicht als rechtsstaatliche Staatstätigkeiten qualifizieren, aber in der Weimarer Reichsverfassung mehr als bloß die Kontinuität des rechtsstaatlichen Paradigmas am Werk sehen. In seinem Kommentar aus den 1950er-Jahren zu *Die Auflösung des Enteignungsbegriffs* schreibt Schmitt (1973, S. 119; Hervorhebungen im Original) vielsagend:

*Rechtsbegrifflich* sind Eigentum und Enteignung in jeder Rechts- und Wirtschaftsordnung einander zugeordnet und strukturell korrespondierende Begriffe, vorausgesetzt, daß die Enteignung ein Rechtsinstitut und nicht irgendeine Entziehung, irgendein Eingriff oder Rechtsverlust sein soll. Eine bürgerlich-rechtsstaatliche Eigentumsordnung hat infolgedessen nicht nur einen anderen Eigentums-, sondern auch einen anderen Enteignungsbegriff wie eine sozialistische oder eine feudale Eigentumsordnung.

Insofern bestätigt Schmitt die zugrundliegende These des vorliegenden Textes. Der spezifische Staatsbegriff bestimmt die Form des jeweiligen Eigentumsbegriffs. Dadurch, dass Schmitt die Weimarer Reichsverfassung primär auf einen – sehr engen materiellen – Rechtsstaatsbegriff reduziert, erhält er die Möglichkeit, das Privateigentum mit einer besonderen Dignität zu versehen und durch eine allein rechtsstaatliche Perspektivierung des Gesetzesbegriffs die bestehende Privateigentumsordnung gegen entschädigungszurückweisende Umverteilungsansprüche zu immunisieren. Die Aporie des demokratischen Rechtsstaates löst Schmitt eindeutig zugunsten des Rechtsstaatlichen auf.

### Otto Kirchheimer: Die „volonté générale“ und ihr Privateigentum

Anders als Carl Schmitt sah der sozialistische Staatstheoretiker Otto Kirchheimer in der Weimarer Reichsverfassung nicht die rechtliche Manifestation einer Entscheidung im Sinne eines Kontinuums bürgerlicher Rechtsstaatlichkeit fortbestehen. Kirchheimer stellt in der Reichsverfassung den verfassungsrechtlichen Ausdruck bloßer Unentschiedenheit fest, einer Unentschiedenheit, die nicht fähig war, auf die virulent gewordene Frage *Bürgertum oder Arbeiterinnenklasse?* eine klare Antwort zu formulieren. Der Klassenantagonismus wurde insbesondere durch die Inkongruenz der Verfassung namentlich des Grundrechtsteils, einer „Ankerreihe“ (Kirchheimer 2017a, S. 230) unterschiedlichster Weltanschauungen, an die Sphäre politischer Durchsetzungs- und Repressionsfähigkeit delegiert. Kirchheimer ging dabei so weit, die in Weimar gängige Chiffre, die *Weimarer Reichsverfassung sei ein „Kompromiss“*, zurückzuweisen. In seinem eminenten Text *Weimar … und was dann?* von 1930 schreibt er (2017a, S. 230) daher:

Unter Kompromiss versteht man gemeinhin eine Lösung, die durch Nachgeben beider Teile gewonnen wird und eine bestimmte Sachlage für eine gewisse Zeitspanne endgültig, eindeutig und abschließend regeln will. Ein solches Nachgeben ist in den Grundrechtsbestimmungen der Weimarer Verfassung nicht erfolgt. Man hat dort vielmehr regelmäßig unter einem dem sozialstaatlichen Wörtervorrat Naumanns entnommenen zierenden Vorspruch oder einleitenden Artikel verschiedene, den entgegengesetztesten Kultur- und Sozialanschauungen entsprechende, Bestimmungen nebeneinandergestellt.

Kirchheimer ist demnach anders als Schmitt nicht darum bemüht, einen kongruenten Verfassungsbegriff seiner Deutung zu unterlegen, sondern hebt im Gegenteil die Ambivalenz und Inkongruenz der Reichsverfassung hervor, um dadurch die der Verfassung unterlegte politische Sphäre in den Blick zu nehmen. Das Konstatieren der Ambivalenz nötigt zur verfassungspolitischen Befragung nach den der Verfassung inhärenten Machtstrukturen – und qualifiziert die Betrachtung dadurch als spezifisch sozialwissenschaftliche (vgl. auch Buchstein 2019, S. 110–111).

Es ist daher nur konsequent, dass Kirchheimer vor allem Perspektivierungen wie die Carl Schmitts als ideenpolitische Interventionen zugunsten des Bürgertums ablehnt und stattdessen mit Nachdruck auf die Widersprüchlichkeit der Verfassung beharrt:

Während die liberale Verfassungsbetrachtung […] eine nicht vorhandene Einheit vortäuscht, um mit ihr alle Zwiespältigkeiten der gegenwärtigen Gesellschaftsorganisation zu verdecken, muss eine sozialistische Verfassungsbetrachtung alle jene Widersprüche aufdecken, die der heutigen Gesellschaftsorganisation und ihrer politischen Form anhaften (Kirchheimer 2017a, S. 209).

Die Sichtbarmachung der verfassungspolitischen Aporien ist vom Anspruch geleitet, die Verfassung primär als Klassenkampf zu deuten und dadurch eine politische Offenheit aufzudecken, die Kirchheimer freilich als Hebel zur Plausibilisierung transformatorischer Potenzen hin zu einer sozialistischen Ordnung nutzt. Kirchheimer betont konsequent die Zäsur durch die Weimarer Revolution, indem er die erste deutsche Republik vor allem als Frage an die zukünftige politische Ordnung dechiffriert.

Die Weimarer Zäsur begreift Kirchheimer dabei nicht als kontingentes Phänomen, sondern stellt die Prozesse seiner Gegenwart in eine größere historische Linie, die in den geschichtsphilosophischen Bahnen sozialistischer Teleologie verläuft. Im Verhältnis zum 19. Jahrhundert sieht der Verfassungstheoretiker im 20. Jahrhundert ein Verblassen der liberal-individualistischen Semantiken durch eine historische Bewegung hin zur amorphen Kategorie der „Masse“ am Werk (vgl. Kirchheimer 2017b, S. 291). Nicht mehr der Kampf zwischen absolutistischer Ordnungsrationalität und liberalem Individualismus dominiere das politische Bewusstsein, sondern die durch geschichtliche und vor allem ökonomische Prozesse generierten Kollektivsubjekte, die auf Integration in die neue Ordnung drängen: „Überdies ist das Gegenstück des Absolutismus, der einzelne angegriffene und schutzwürdige Bürger, im Zeitalter der Verbände kaum mehr vorhanden, so dass diese Bestimmungen in dem Maße, wie sie uns selbstverständlich geworden sind, an Bedeutung verloren haben“ (Kirchheimer 2017b, S. 231). Der „Kollektiv“- oder „Verbandsmensch“ des Klassenkampfes löste das abstrakte Individuum des Liberalismus als hegemoniale Figur ab (vgl. auch Müller 2018, S. 17–84). So heißt es in Kirchheimers für die Enteignungsdebatte eminenten und ebenfalls wie *Weimar … und was dann?* 1930 erschienenen Text *Die Grenzen der Enteignung*: „Für diesen kollektivierten Menschen, der im Rahmen fester ökonomischer Bindungen lebt und tätig ist, muss die neue Verfassung nicht nur grundsätzlich Freiheiten im alten Sinn […] enthalten, sie muss auch positiv seiner Tätigkeit Raum geben, ihre Wirkkraft nicht nur anerkennen, sondern fördern“^11^ (Kirchheimer 2017b, S. 289).

Vor diesem Hintergrund kann Kirchheimers Darstellung des Artikels 153 nur auf die Markierung einer Abwendung vom 19. Jahrhundert, initiiert durch den gesellschaftlichen Wandel im Zeitalter der Kollektivität, hinauslaufen, um das emanzipatorische Potenzial zugunsten des Proletariats offenzulegen. Die Eigentumsbestimmung der Reichsverfassung changiere im besonderen Maße, so Kirchheimer, im bipolaren Spannungsfeld zwischen Bürgertum und Arbeiterinnenklasse: „Der Artikel 153 der Reichsverfassung verdankt seine Einzelausgestaltung ebenso sehr verfassungsrechtlichen Reminiszenzen aus dem vergangenen Jahrhundert, wie den sozialen Bedürfnissen der Neuzeit“ (Kirchheimer 2017b, S. 293).^12^ Zum einen bleibt das Recht auf Privateigentum weiterhin bestehen als ein negatives Freiheitsrecht des Individuums und ist damit nach wie vor an die individuelle Willkürsphäre verwiesen. Andererseits fordert der Einzug der Kollektivität in die gesellschaftliche Evidenz die Abstraktion des Individuums heraus, indem die ursprüngliche Transzendierung der Einzelnen zum rechtlich konstruierten kontextlosen Individuum wieder zurück in die soziale Eingebundenheit geholt wird. Für die Eigentumsbestimmung bedeutet dies eine Infragestellung der individuellen Willkürsphäre durch die soziale Situiertheit der Einzelnen als in Kollektiven handelnder Mensch. Es kann demnach nicht mehr darum gehen, das Privateigentum als negatives Freiheitsrecht des Individuums zum Kern des Staates zu erheben, sondern muss durch den Wandel des individuellen zum kollektiven Paradigma stets in sozialen Zusammenhängen ausgeleuchtet werden. Dies scheint für Kirchheimer gerade in der Weimarer Reichsverfassung berücksichtigt worden zu sein:

Die Weimarer Reichsverfassung hat am deutlichsten von allen Nachkriegsverfassungen das laissez-faire, laissez-passer, das die bürgerlichen Verfassungen des 19. Jahrhunderts den Fragen der Wirtschaft gegenüber bezeugten, endgültig beseitigt. Sie hat den Willen gezeigt, damit aufzuräumen, die wirtschaftliche Betätigung des Menschen in den Bereich einer sie nicht interessierenden Freiheit zu verweisen. Sie garantiert nicht mehr nur, sie will selbst verantwortlich sein (2017b, S. 287).

Im Kontrast zum Paradigma des auf bürgerlicher Freiheit und Privateigentum bauenden liberalen Rechtsstaates hebt Kirchheimer die revolutionäre Irritation der Hermetik von Staat und Individuum in Weimar hervor: „Die Stützen der kapitalistischen Wirtschaftsordnung: Vertragsfreiheit, Privateigentum und Erbrecht haben ihre bisherige Unnahbarkeit aufgeben müssen. Von Rechten, die dem Staat starr und unabänderlich gegenüberständen, kann keine Rede sein“ (Kirchheimer 2017b, S. 288).

Mit Blick auf den sich ereigneten Bruch in der Ausgestaltung der Eigentumsbestimmungen macht Kirchheimer vom kritischen Instrument der „erworbenen Rechten“ im postrevolutionären Weimar Gebrauch. Unter erworbenen Rechten möchte Kirchheimer Rechte verstanden wissen, „deren Entstehungstatbestand in einer überholten Sozialordnung begründet liegt“ (Kirchheimer 2017b, S. 273). Das Erbe Ferdinand Lassalles affirmierend als „richtig und zeitlos gültig“ (Kirchheimer 2017b, S. 277) annehmend, lautet für Kirchheimer (2017b, S. 274; Hervorhebungen im Original) die wesentliche Fragestellung: „*Welche Rechte sind gegen Entschädigung, welche Rechte ohne Entschädigung aufzuheben.*“ Der revolutionäre Bruch stellt unmittelbar die bestehende Ordnungsstruktur mit den sie stützenden Selbstverständlichkeiten infrage und macht den Konstruktionscharakter rechtlicher Regime virulent. Die Unentschiedenheit der Reichsverfassung lässt im Paradigma des Klassenkampfes die Richtungsentscheidung offen, sodass Kirchheimer (2017b, S. 276) im Anschluss an Lassalle polemisch-interventionistisch ausführt:

Jede Stellungnahme, die die Frage [nach den erworbenen Rechten; M. K.] so lösen will, dass sie die Gebote einer Willensmacht, die vergangenen Zeiten angehört, als für die Gegenwart und Zukunft heilig und unabänderlich hinstellt, hat Lassalle zurückgewiesen. Das Individuum ist souverän im Erwerb von Rechten, solange die Gesetzgebung den betreffenden Rechtsinhalt zu erwerben gestattet. Dadurch erhält das Individuum aber keineswegs das Recht, sich als Gesetzgeber für die Zukunft zu proklamieren und jeder neuen Gesetzgebung den Schein seines Rechts entgegenzusetzen.

Es ist eine Frage des „gegenwärtigen Zeitbewusstseins“ (Kirchheimer 2017b, S. 277), das Kirchheimer institutionell im Parlament realisiert sieht (vgl. Kirchheimer 2017b, S. 278), welche Rechte vergangener Ordnung weiterhin gelten dürfen.

Die Überlegungen zur Historizität des Rechts im Begriff der erworbenen Rechte bilden für Kirchheimers Schrift *Die Grenzen der Enteignung* den Ausgangspunkt einer Kritik an juristischen Verteidigungen der bestehenden Eigentumsordnung. Unschwer ist mit der für die erworbenen Rechte maßgeblichen Frage nach der Entschädigungsnotwendigkeit eines Rechtentzugs ein Verweis auf die für Weimar entscheidende Enteignungsunbestimmtheit erkennbar: Darf das Recht auf Privateigentum entschädigungslos entzogen werden? Die Idee erworbener Rechte wird von Kirchheimer als ideenpolitisches Instrument für eine Infragestellung der bestehenden Privateigentumsordnung umgedeutet, um einen begrifflichen Beitrag zur Frage im kollektiven Zeitalter des Klassenkampfes zugunsten des Proletariats zu leisten. Insbesondere die Ambivalenz in den Enteignungsbestimmungen des Artikels 153 Absatz 2 werden dabei hervorgehoben und die Potenzialität entschädigungsloser Eingriffe in das Privateigentum qua Reichsgesetz stark gemacht. In *Die Grenzen der Enteignung* führt Kirchheimer konsequent in den Bahnen des bisherigen Deutungsschemas aus:

Aber auch die individuelle Eigentumsgarantie des Absatz 2 [des Artikels 153; M. K.] wurde noch in sich abgeschwächt dadurch, dass man die angemessene Entschädigung durch ein Reichsgesetz für ausschließbar erklärte. Damit durchbricht die Verfassung auch die individuellen Eigentumsgarantie und betont nochmals, dass der Enteignung nicht mehr ihr Ergänzungscharakter im Verhältnis zur Eigentumsgarantie zukommt, sondern dass sie lediglich ein Unterfall des allgemein ausgesprochenen Grundsatzes ist: Dem Gesetzgeber gegenüber kann sich der Eigentümer nicht auf die Eigentumsgarantie berufen (Kirchheimer 2017b, S. 298).

Durch die Möglichkeit, eine Entschädigung qua Reichsgesetz zu umgehen, sieht Kirchheimer die Annahme eines grundlegenden Wandels der Weimarer Reichsverfassung gegenüber der Rechtsstaatsrationalität des 19. Jahrhunderts bestätigt. Die Garantie des Eigentums wird nicht als unverletzbares Naturrecht hypostasiert und durch den klassischen Enteignungsbegriff abgesichert, sondern der institutionellen Sphäre der Gesetzgeberin untergeordnet. Zwar besteht weiterhin ein generelles Recht auf Eigentum, da dieses grundrechtlich „gewährleistet“ ist, doch wird es zugleich der Definitionsmacht der Gesetzgeberin zugesprochen, die „Inhalt und Schranken“ bestimmt: „Solange es eine Kategorie Eigentum gibt, bedeutet Eigentum ein absolutes Herrschaftsrecht, absolut allerdings nur in der Sphäre des Privatrechts und unterworfen der Souveränität des Staates und damit der gesetzgebenden Körperschaft“ (Kirchheimer 2017b, S. 296). Otto Kirchheimer erhebt dadurch die Gesetzgeberin zum zentralen Akteur, die vor dem Hintergrund des Theorems erworbener Rechte im postrevolutionären Weimar frei über die Reichweite des Privateigentums verfügen und nicht durch grundrechtliche Beschränkungen gehegt werden kann. Die Potenzialität qua Gesetz entschädigungslos in das Privateigentum einzugreifen, wird von Kirchheimer als Souveränität des Volkes umgedeutet, das durch Parlamentsmehrheiten selbst darüber zu Gericht sitzt, welche erworbenen Rechte weiterhin und in welchem Umfang gelten und welche entschädigungslos durch souveräne Entscheidung negiert werden.

An diesem Punkt ist ersichtlich, wie Kirchheimer den von Schmitt eingeworfenen Generalitätsimperativ des Rechtsstaates durch eine demokratische Rückbindung an den souveränen Volkswillen unterläuft. Mit dem Verblassen der bürgerlichen Rechtsstaatsrationalität, wie sie Schmitts Gesetzesbegriff bemüht, ist zugleich die gesetzliche Bindung des Souveräns zugunsten der Einzelnen umgangen, da nicht mehr der Schutz des abstrakten Individuums der Nukleus des Staates ist, sondern die Souveränität des sich im Gesetz materialisierenden Volkes. Die verfassungsrechtliche Unentschiedenheit ist die Hinwendung zum demokratischen Kampf um parlamentarische Mehrheiten, die um die Ausgestaltung der Verfassung ringen. Explizit gegen Schmitt schreibt Kirchheimer (2017b, S. 302) daher programmatisch:

Die Reichsverfassung hat aber die erworbenen Rechte und ihre Eingliederung in den Staat in das Gebiet der Gesetzgebung verwiesen. Da sie eine demokratische Verfassung ist, hat sie diese Verweisung nicht nur, wie Carl Schmitt meint, für generelle Beschränkungen des Eigentums vorgenommen, sie hat dem Gesetzgeber auch in der Setzung individueller Enteignungsakte freie Hand gelassen. Wenn die verfassungsmäßige Zulässigkeit individueller gesetzlicher Enteignungsakte damit bekämpft wird, dass man den generellen Charakter des Gesetzes als notwendiges rechtsstaatliches Postulat bezeichnet, so mag an dieser Stelle dahingestellt bleiben, ob genereller Gesetzescharakter wirklich grundlegende Voraussetzung für den Rechtsstaat ist, entschieden bestritten muss aber werden, dass für die Demokratie ebenfalls die Lehre von der notwendigen Beschränkung des Gesetzes auf generelle Tatbestände Gültigkeit haben kann.

Die Generalität des Gesetzes wird von Kirchheimer als ideenpolitische Hegung der parlamentarischen Gesetzgeberin umgedeutet, die die Kontinuität des rechtsstaatlichen Paradigmas als Vehikel zur Zurückweisung von demokratischen Umstrukturierungsversuchen der Privateigentumsordnung nutzt. Kirchheimers Blick ist damit ein primär demokratischer, der die Souveränität des Volkes als rechtssetzende Instanz perspektiviert: „Das generelle Moment des demokratischen Gesetzes liegt in seinem Ursprung, nicht in seiner Tendenz beschlossen“ (Kirchheimer 2017b, S. 302). Die „volonté générale“ wird damit der inappellable Wille, der qua Mehrheit im Parlament in Gesetzesform entschädigungslos in das Privateigentum eingreifen und darüber entscheiden kann, welche erworbenen Rechte übernommen werden sollen.

Es zeigt sich nun auch deutlich bei Kirchheimer, dass der Begriff des Staates den Begriff des Eigentums bestimmt. Während Schmitt durch die Generalität des Gesetzes das Moment des Rechtsstaates in Weimar adressiert und auf diese Weise in der Kontinuität des Konstitutionalismus operierend die negative Freiheit des Individuums sowie das Privateigentum zum Nukleus der Rationalität erhebt, stellt Kirchheimers Argumentation auf das Revolutionäre der ersten deutschen Demokratie ab und unterstreicht die demokratische Quelle des Gesetzes, die „volonté générale“, als einzigen Maßstab. Durch die differierenden Staatsbegriffe werden unterschiedliche Enteignungspotenzialitäten aufgerufen und insofern auch unterschiedlich die jeweilige Eigentumsordnung beschrieben. Zwischen den heuristischen Polen bürgerlicher Rechtsstaat oder Demokratie positioniert sich Kirchheimer auf der Seite des Demokratischen.

## Resümee: Der demokratische Rechtsstaat und die unbestimmte Sonderstelle des Privateigentums

Die Eigenheit eines revolutionären Umbruchs ist das Stellen von Fragen: Mit welcher Ordnung hat man es künftig zu tun? Wie ist das Verhältnis zur überwundenen Ordnung? Welche Begriffe können übernommen werden und welche verlieren ihren Sinn? Politische Umbruchserfahrungen fordern insofern ein erhöhtes Maß theoretischer Anstrengung, als die Selbstverständlichkeiten des Alltäglichen grundlegenden Erschütterungen ausgesetzt sind. Die Weimarer Revolution ist dafür ein besonderes Beispiel, da 1918/19 Kontinuität und Bruch im unbestimmten Begriff des demokratischen Rechtsstaates kulminierten: Zwischen den beiden Polen *Rechtsstaat und Demokratie* und ihren jeweils adressierten Subjekten *Individuum und Volk* changierend – so ist zu resümieren – war die Ausmessung der neuen aporetischen Ordnung Aufgabe des politischen Denkens, das Begriffe wie Volk, Individuum, Eigentum und Staat zu koordinieren hatte. Die Weimarer Gegenwart forderte ein politisches Denken, das in besonderer Intensität den Dialog mit der Gegenwart zu suchen hatte, um den drängenden Fragen zu begegnen und vermittels Begriffe auf die Ausgestaltung der zukünftigen Ordnung Einfluss zu nehmen.

Der auf herausgehobene Weise die Konflikte um die neue Ordnung konzentrierende Begriff des Privateigentums ist vor diesem Hintergrund nicht allein Ausgangspunkt einer Diskussion ökonomischer oder privatrechtlicher Art, sondern veranlasste vielmehr ein „Tauziehen“ um die Spezifik der Republik. Die Frage nach dem Ort des Eigentums in der Architektur des demokratischen Rechtsstaates war gleichbedeutend mit der Frage nach dem demokratischen Rechtsstaat selbst, da der Begriff des Privateigentums seine konkrete Ausgestaltung erst vor dem Hintergrund einer Theorie des Staates erfuhr. Die Praxis der Enteignung als Schnittstelle zwischen Individuum, Eigentum, Staat diente als begrifflicher „Prüfstein“ (Kirchheimer 2017b, S. 272), dieses Verhältnis zu vermessen. Carl Schmitt und Otto Kirchheimer können in Weimar idealtypisch als Antipoden im Lokalisierungsversuch des Privateigentums beschrieben werden, die entweder wie Carl Schmitt das begriffliche Gewicht auf den bürgerlichen Rechtsstaat oder wie Otto Kirchheimer auf das Element der Demokratie legend in ihren Fragen um die Reichweite von Enteignungspotenzialitäten nicht allein die Rechtslage, sondern vielmehr die Staatsordnung als Ganzes untersuchten.

Dabei scheint die dargestellte Ambivalenz der Eigentumsdiskussionen im Spannungsfeld zwischen Demokratie und bürgerlichem Recht nicht allein ein rein ideenhistorisches Problem zu sein, sondern eine begriffsimmanente Aporie methodisch sichtbar zu machen, die den demokratischen Rechtsstaat systematisch bestimmt und bis in die Gegenwart wiedererkennbare Muster zeitigt. Die theoretische Unbefangenheit der Autorinnen Weimars kann dabei helfen, „die Eindimensionalität der Gegenwart zu irritieren“ (Maus 2018, S. 204). Ausblickend könnte daher das Weimarer Ringen um den Ort des Privateigentums im demokratischen Rechtsstaat Anstöße geben, gegenwärtige Eigentums- beziehungsweise Enteignungsfragen im heuristischen Muster zwischen Demokratie und bürgerlichem Rechtsstaat zu rahmen, um auf diese Weise einen begrifflichen Zugang zu Eigentumskonflikten für die Politische Theorie zu eröffnen.

## Footnotes

1 Der Beitrag verwendet das generische Femininum, um auf alle Geschlechter zu verweisen.

2 Die fehlende Aufmerksamkeit der Politischen Theorie für Enteignungsfragen hängt mit einem allgemeinen Defizit an eigentumstheoretischen Überlegungen zusammen (vgl. Hacke 2018, S. 294), sodass Tilo Wesche (2014, S. 444) der Demokratietheorie gar eine „Eigentumsvergessenheit“ attestiert. Zwar kann eine allmähliche Veränderung dieses Umstandes nicht zuletzt aufgrund des Welterfolges von Thomas Piketty (2014) und John Rawls’ Konzept der *Property Owning Democracy* (vgl. Cheneval und Lazlo 2013) vor allem in der Soziologie und Sozialphilosophie (vgl. Wesche 2014; Rosa und Wesche 2018) und (von Piketty und Rawls unabhängig) im Feld der kritischen Rechtsphilosophie (vgl. Menke 2018, S. 207–225; Loick 2018) konstatiert werden, doch bleibt der auffällige Mangel insgesamt bemerkenswert, wenn berücksichtigt wird, welchen Stellenwert das Privateigentum in der Ideengeschichte einnimmt (vgl. Macpherson 2016). Für den englischsprachigen Raum sind die in einer rechtsphilosophischen Tradition stehenden anhaltenden Bemühungen von James Penner (1997, 2013, 2020; Penner und Otsuka 2018) hervorzuheben. Eine Analyse der Literatur zum Enteignungsbegriff zeigt deutlich, dass das Institut nach wie vor überwiegend im Zusammenhang der Verwaltungsrechtswissenschaft als „klassische Enteignung“ thematisiert wird. Als ideenpolitisches Projekt ist die Reihe *Bibliothek des Eigentums *der *Deutschen Stiftung Eigentum* ferner zu thematisieren, die vor allem programmatisch einen interventionistischen Ansatz zugunsten des Privateigentums verfolgt (vgl. Deutsche Stiftung Eigentum 2021). In diesem Zusammenhang stehend sind insbesondere der historisch-vergleichende Sammelband von Otto Depenheuer und Foroud Shirvani (2018) *Die Enteignung* sowie der Sammelband von Depenheuer (2014): *Staatssanierung durch Enteignung? *herauszustellen.

3 Exemplarisch seien hier bloß die wichtigsten Texte der Debatte genannt. Verfassungskommentare und Lehrbücher werden, um die Übersichtlichkeit zu wahren, ausgespart. Den Auftakt machte der den ökonomischen Status quo grundlegend verteidigende, dabei viel beachtete sowie prägende Text des Zivilrechtlers Martin Wolff (1923) *Reichsverfassung und Eigentum* von 1923. Es folgte das politisch ähnlich ausgerichtete, sowohl für die Enteignungsdiskussion als auch für den „Methoden- und Richtungsstreit“ eminente Rechtsgutachten von Heinrich Triepel (1924) *Goldbilanzverordnung und Vorzugsaktien* von 1924. Einen historischen Kristallisationspunkt der enteignungstheoretischen Bemühungen bildete der Streit um das Ver﻿mögen der depossedierten Fürsten, der 1926 die Öffentlichkeit bestimmte und als „Fürstenenteignung“ firmierte (vgl. dazu Stentzel 2000; Schüren 1978). In diesem Zusammenhang sind der später noch detailliert zu diskutierende Text *Die Auflösung des Enteignungsbegriffs* von 1929 (Schmitt 1973) sowie das politisch höchst wirksame Rechtsgutachten von Carl Schmitt (1926) *Unabhängigkeit der Richter, Gleichheit vor dem Gesetz und Gewährleistung des Privateigentums nach der Weimarer Verfassung* von 1926 zu nennen, die jeweils auf die Zementierung des ökonomischen Status quo abstellten. Ebenso wie die zuvor genannten Texte von Wolff, Triepel und Schmitt versucht ferner auch Walter Schelcher (1930) in seinem Text *Gesetzliche Eigentumsbeschränkung und Enteignung* ideenpolitisch einen umfassenden Schutz der bestehenden Eigentumsordnung zu plausibilisieren. Eine andere politische Richtung schlugen dagegen zum einen der weiter unten noch besprochene Text von Otto Kirchheimer (2017b) *Die Grenzen der Enteignung* von 1930 sowie – ebenfalls 1930 erschienen – *Reichsverfassung und Eigentum* (2017c) und Franz L. Neumanns (1978a) verfassungstheoretische Überlegungen in *Die soziale Bedeutung der Grundrechte in der Weimarer Verfassung* und in *Über die Voraussetzungen und den Rechtsbegriff einer Wirtschaftsverfassung *(1978b) ein.

4 Wenn hier von Staatswissenschaften gesprochen wird, soll auf den Umstand aufmerksam gemacht werden, dass eine disziplinäre Trennung zwischen Politik- und Rechtswissenschaft insbesondere des Staatsrechts in Weimar schwer zu ziehen ist. Da die Politikwissenschaft in Deutschland noch nicht im ausreichenden Maß institutionalisiert war, wurden viele aus heutiger Sicht eher der Politikwissenschaft zuzuordnende Problemstellungen von Weimarer Juristen behandelt. Insbesondere Autoren des ‚geisteswissenschaftlichen Lagers‘ beziehungsweise die ‚Antipositivisten‘ im ‚Methoden- und Richtungsstreit‘ wie Rudolf Smend, Carl Schmitt, Heinrich Triepel und Hermann Heller sind hier hervorzuheben, da sie immer auch – oder gerade – die der Rechtsordnung unterlegte politische Sphäre als Geltungsgrund des Rechts thematisierten (vgl. Heun 1989; Friedrich 1997, S. 320–330).

5 Eine duale Struktur des Grundrechtsteils zeigt sich vor allem in Wirtschaftsfragen und dient eher als Heuristik für die weitere Untersuchung. Sie ist damit nicht für den gesamten Grundrechtsteil plausibilisierbar. Beispielsweise stehen die religionspolitischen Forderungen, die das Zentrum in den Grundrechtskatelog einbrachte, quer zu der Klassenkampffolie. Insbesondere die intensive Diskussion über die verfassungsrechtlich sanktionierte Zulassung konfessionell gebundener Schulen fügt sich nicht dem Klassenkampfmuster (vgl. Winkler 2019, S. 104). Die Folie des Klassenkampfes dient an dieser Stelle zur Konturierung der weiter unten thematisierten Eigentumsproblematik.

6 Innerhalb der Kant-Deutung ist umstritten, wie weit Kants eigentumstheoretische Exklusionsdimension reicht. Richard Saage (1998) unterscheidet primär zwischen zwei Lesarten: einer besitzindividualistischen, die den Schutz des Eigentums zur zentralen Aufgabe des Staats erhebt und allein Formen der Armenfürsorge als minimale Praxis staatlicher Intervention zulässt (vgl. Saage 1973; Dreier 1991; Zotta 2000), und einer wohlfahrtsstaatlich-konsensualen, die vor allem die soziale Verfasstheit des Eigentums als Eigentums*recht* betont (vgl. Luf 1978; Kühl 1984). Ausgangspunkt der Kontroverse ist die Bedeutung des jeweiligen sozialen Aggregationszustands der von Kant bemühten kontraktualistischen Dichotomie von Natur- und Gesellschaftszustand für die Form des Eigentumsrechts. Wird der Naturzustand stärker hervorgehoben, so ist das staatlich organisierte Eigentumsrecht eine reaktive rechtliche Kodifikation einer durch Okkupation zustande gekommenen Inbesitznahme; wird dagegen der Kontrakt als Modus des *allgemeinen Willens* fokussiert, können sozialstaatliche Interventionen in bestehende Eigentumsordnungen durch den Hinweis auf die konsensuale Bedingung plausibel gemacht werden. Unabhängig davon, inwiefern Kant Formen staatlich angeleiteter proprietärer Distribution zulässt, wird Saage (1998, S. 246) zugestimmt, wenn dieser schreibt: „Niemand kann bestreiten, daß die Funktion der Eigentumssicherung nicht nur bei Locke, sondern auch bei Kant im Zentrum der Staatsaufgabenlehre der ‚Metaphysik der Sitten‘ steht.“ Die kantische Idee der Gleichheit ist primär eine formal-rechtliche und keine substanzielle. Da es sich im Folgenden um eine schematisch verfahrende Konturierung eines spezifischen Arguments und Darstellung einer diskursiven Ausgangssituation handeln soll, werden philologische Diskussionen nur vereinzelt aufgegriffen.

7 Die Konfliktlinie kann freilich nicht nur in der Sprache des Klassenkampfes formuliert werden, sondern findet sich gegenwärtig beispielsweise in Diskussionen um die demokratische Integration von Frauen wieder. Fiona Mackay (2008) sieht in der Umverteilung („redistribution“) einen entscheidenden Hebel zur adäquaten Berücksichtigung von femininen Positionen im demokratischen Prozess. Zum Verhältnis von „Umverteilung“ und „Anerkennung“ ist freilich noch Nancy Fraser (2021) hervorzuheben.

8 Es kann vor diesem Hintergrund bestätigend angeführt werden, dass Carl Schmitt zwar in der Weimarer Verfassung insbesondere im Grundrechtsteil „dilatorische Formelkompromisse“ eingeschrieben sah, also Kompromisse, die „nicht die sachliche Erledigung einer Frage im Wege beiderseitigen sachlichen Nachgebens [betreffen], sondern [deren; MK] Einigung [dahingeht], sich mit einer dilatorischen Formel zu begnügen, die allen Ansprüchen Rechnung trägt“ (Schmitt 2017, S. 32). Die von ihm angeführten Beispiele für Formelkompromisse betreffen allerdings den dritten und vierten Abschnitt der Grundrechte und Grundpflichten, die das Verhältnis Staat und Schule sowie Staat und Kirche regeln. Für die staatliche Ordnung gilt Carl Schmitt zufolge klar: „Enthielte die Weimarer Verfassung nichts als solche dilatorischen Kompromisse, so wäre ihr Wert allerdings illusorisch, und man müßte darauf gefaßt sein, daß sie die grundlegenden Entscheidungen *außerhalb* der verfassungsgesetzlich vorgesehenen Verfahren und Methoden fallen. Darin aber, daß sie die grundlegenden politischen Entscheidungen über die politische Form und die Prinzipien des bürgerlichen Rechtsstaates klar und eindeutig trifft, liegt – politisch wie juristisch – die Substanz der Weimarer Verfassung“ (Schmitt 2017, S. 35; Hervorhebungen im Original). Dadurch bleibt Schmitt in einer konstitutionalistischen Deutungstradition, mit dem Unterschied, dass nicht mehr von einer konstitutionellen Monarchie, sondern nun von einer konstitutionellen Demokratie gesprochen werden muss.

9 Die diskursive Nähe zwischen Kant und Schmitt hinsichtlich des Verhältnisses von Rechtsstaat und Eigentum darf nicht insofern missverstanden werden, als suche Schmitt den Rechtsstaat dadurch gegen aller Erwartung zu verteidigen. Dass die hier ausgeführte Nähe zu Kant von Schmitt von einem polemischen Motiv getragen ist und von Beginn an auf eine Unterminierung des Weimarer Rechtsstaats durch einen zum Scheitern angelegten Begriff desselben abzielt (vgl. zur methodischen Polemik Schmitts Neumann 1988), wird im Weiteren noch deutlich zu machen sein (vgl. Fußnote 10).

10 Carl Schmitt bemüht hier den in Weimar insbesondere unter Gegnern der Republik verbreiteten Topos des „Parlamentsabsolutismus“, den er durch eine extensive Interpretation der Grundrechte im Medium des Rechts zu hegen versucht (vgl. auch Stolleis 1999, S. 111). Er positioniert sich mit der Argumentation für die Gesetzesbindung der Gesetzgeberin im zeitgenössischen Diskurs des Weimarer Staatsrechts. Im „Methoden- und Richtungsstreit“ wurde die Frage um die Gesetzesbindung eine der Hauptkonfliktlinien der Disziplin (vgl. VVDStRL 3 1927, S. 2–48). Die Gesetzesbindung avancierte zu einem willkommenen Instrument, um die parlamentarische Gesetzgeberin in ihrer Handlungsfähigkeit einzuschränken, weshalb gerade parlamentarismuskritische Staatsrechtslehrer auf unterschiedliche Weise die Grundrechte gegenüber der Legislative ausspielten (vgl. Heun 1989). Dadurch ist zugleich auch gesagt, dass Schmitts enger Zuschnitt des Rechtsstaatsbegriffs nicht von einem Motiv der Stärkung des Rechtsstaats in Weimar getragen ist, sondern das polemische Vehikel wird, um zum einen die parlamentarische Handlungsfähigkeit juristisch zurückzudrängen und zum anderen die Eigentumsverhältnisse unangetastet zu lassen. In seinem programmatischen Vortrag *Starker Staat und gesunde Wirtschaft* (1995, S. 75; Hervorhebungen im Original) von 1932 macht Schmitt diesen Anspruch unmissverständlich deutlich: „Der heutige deutsche Staat ist *total aus Schwäche *und Widerstandslosigkeit, aus der Unfähigkeit heraus, dem Ansturm der Parteien und der organisierten Interessenten stand zu halten.“ Anstatt als technischer Apparat allein zu einem Hebel parteipolitischer Partikularinteressen herabzusteigen, fordert Schmitt zur Entschiedenheit auf, „sich von nichtstaatlichen Dingen abzusetzen“ (Schmitt 1995, S. 71). Einen idealisierten faschistischen Staat imaginierend sei das primäre Ziel übergeordnet autoritär-personal, daher antipluralistisch, zu agieren und, sofern ökonomische Prozesse nicht die Sphäre des staatlichen Machterhalts tangieren, in organisierender Nichtbeachtung über dem zu stehen, was Schmitt homogenisierend als „Wirtschaft“ bezeichnet. Dabei sei die liberale Dichotomie von Staat und Gesellschaft insofern unhaltbar, als der Staat in industrialisierten funktional ausdifferenzierten Gesellschaften nicht allein der Wirtschaft gegenüberstehen könne, sondern stets intervenieren müsse, diese Intervention allerdings nicht sozialstaatliche Intentionen verfolgen solle, sondern primär der Aufrechterhaltung einer „Sphäre der freien Wirtschaft“ (Schmitt 1995, S. 81) diene. Den Bedeutungsgehalt der bereits im Titel angesprochenen „gesunden“ oder „freien Wirtschaft“ klärt Schmitt nicht auf (vgl. auch Hacke 2018, S. 344–345). Sein Begriff des Rechtsstaats richtet sich daher, um dies nachdrücklich zu betonen, gegen den Rechtsstaat selbst: Durch einen materiellen Begriff des Gesetzes soll der parlamentarische Aktionsradius in Weimar – insbesondere gegen sozialistische Gestaltungsansprüche gerichtet – möglichst eingeschränkt, während die exekutiven Diktaturbefugnisse des Reichspräsidenten ideenpolitisch als fester Ordnungsgarant für wirtschaftliche Organisation gestärkt werden (vgl. Schmitt 1995, S. 78–79). Insofern liefe man Gefahr, Schmitts polemischen Subtext zu überlesen, wenn man diese „rechtsstaatliche“ Enteignungskritik tatsächlich einem liberalen Register zuordnen und frei von den sonstigen begrifflichen Interventionen insbesondere zugunsten einer autoritären Expansion der Exekutivgewalt lesen würde. Für diesen Hinweis danke ich einer der beiden anonymen Gutachterinnen.

11 Kirchheimer nimmt hier eine These auf, die er bereits in seiner Dissertation (vgl. zusammenfassend Kirchheimer 1928, S. 596) vertrat und die in einer Kritik an einer bloß „formalen Demokratie“, das heißt einer Demokratie, die allein Rechte distribuiert, ohne ökonomische Ungleichheiten anzutasten, mündet. Insbesondere die unter Weimarer Sozialisten prominente Unterscheidung von *politischer* und *sozialer Demokratie* des Austromarxisten Max Adlers ist hier aufgerufen, der zufolge die politische Demokratie aufgrund des Beibehaltens sozioökonomischer Ungleichheiten stets antidemokratische Effekte zeitigt sowie prekär bleibt, wohingegen erst die soziale Demokratie, als Demokratie, die die Klassenunterschiede beseitigt, eine tatsächliche Verwirklichung der Demokratie darstellt (vgl. Buchstein 2017, S. 26–27).

12 Hubertus Buchstein (2017, S. 61) betont, dass Kirchheimer in *Die Grenzen der Enteignung* „die von ihm zuvor in seinen Aufsätzen zum Verfassungstag herausgestellte Widersprüchlichkeit der Weimarer Verfassungsbestimmungen an dieser Stelle nicht wiederholt.“ Es ist Buchstein dahingehend zuzustimmen, dass Kirchheimer durch die Hinweise auf den „Gesamtkomplex“ (Kirchheimer 2017a, S. 288) und „Willen der Verfassung“ (Kirchheimer 2017a, S. 287) anders als beispielsweise in *Weimar … und was dann?* den Eindruck erweckt, der Reichsverfassung sei eine eindeutige Entscheidung vorausgegangen. Doch gerade mit Blick auf seine Deutung des Artikels 153 lässt sich zumindest die sonst von Kirchheimer unterstellte Unentschlossenheit sichtbar machen. Indem Kirchheimer den Artikel 153 zwischen Vergangenheit und sozialistischer Zukunft oszillieren lässt, reproduziert er im Kern die Unentschiedenheitsannahme. Kirchheimers Homogenitätsbegriffe wie „Gesamtkomplex“ und „Willen der Verfassung“ könnten als polemische Begriffe dechiffriert werden, die Einheitlichkeit der Verfassungsentscheidung unterstellen, um sozialistische Transformationsbemühungen verfassungsrechtlich zu plausibilisieren.
